# Deep-Learning-based Automated Identification of Ventriculoperitoneal-Shunt Valve Models from Skull X-rays

**DOI:** 10.1007/s00062-024-01490-4

**Published:** 2025-01-14

**Authors:** Marius Vach, Daniel Weiss, Vivien Lorena Ivan, Christian Boschenriedter, Luisa Wolf, Thomas Beez, Björn B. Hofmann, Christian Rubbert, Julian Caspers

**Affiliations:** 1 Department of Diagnostic and Interventional Radiology, Medical Faculty and University Hospital Düsseldorf, Heinrich-Heine-University Düsseldorf, Moorenstraße 5, 40225 Düsseldorf, Germany; 2 Department of Neurosurgery, Medical Faculty and University Hospital Düsseldorf, Heinrich-Heine-University Düsseldorf, Düsseldorf, Germany

**Keywords:** Ventriculoperitoneal Shunt, X‑Ray, Deep Learning, Computational Neural Networks, Hydrocephalus

## Abstract

**Introduction:**

Ventriculoperitoneal shunts (VPS) are an essential part of the treatment of hydrocephalus, with numerous valve models available with different ways of indicating pressure levels. The model types often need to be identified on X‑rays to assess pressure levels using a matching template. Artificial intelligence (AI), in particular deep learning, is ideally suited to automate repetitive tasks such as identifying different VPS valve models. The aim of this work was to investigate whether AI, in particular deep learning, allows the identification of VPS models in cranial X‑rays.

**Methods:**

959 cranial X‑rays of patients with a VPS were included and reviewed for image quality and complete visualization of VPS valves. The images included four VPS model types: Codman Hakim (*n* = 774, 81%), Codman Certas Plus (*n* = 117, 12%), Sophysa Sophy Mini SM8 (*n* = 35, 4%) and proGAV 2.0 (*n* = 33, 3%). A Convolutional Neural Network (CNN) was trained using stratified five-fold cross-validation to classify the four VPS model types in the dataset. A finetuned CNN pretrained on the ImageNet dataset as well as a model trained from scratch were compared. The averaged performance and uncertainty metrics were evaluated across the cross-validation splits.

**Results:**

The fine-tuned model identified VPS valve models with a mean accuracy of 0.98 ± 0.01, macro-averaged F1 score of 0.93 ± 0.04, a recall of 0.94 ± 0.03 and a precision of 0.95 ± 0.08 across the five cross-validation splits.

**Conclusion:**

Automatic classification of VPS valve models in skull X‑rays, using fully automatable preprocessing steps and a CNN, is feasible. This is an encouraging finding to further explore the possibility of automating VPS valve model identification and pressure level reading in skull X‑rays.

**Supplementary Information:**

The online version of this article (10.1007/s00062-024-01490-4) contains supplementary material, which is available to authorized users.

## Introduction

A hydrocephalus is a condition of increased volume of cerebrospinal fluid (CSF) leading to widening of the intracranial CSF spaces [1]. It can result in neurological symptoms, like headaches, emesis, vision disturbances, loss of consciousness and even death. Hydrocephalus can arise from various causes, including congenital malformations and tumors (which lead to occlusive hydrocephalus), or infections (which typically result in malresorptive hydrocephalus). With a prevalence of approximately 85 in 100,000 globally its socioeconomic impact is substantial, encompassing not only the direct medical costs of treatment and long-term care but also the indirect costs associated with lost productivity and reduced quality of life for affected individuals and their families [2].

A common therapy is the implantation of a ventriculo-peritoneal shunt (VPS). This is a catheter-system implanted between one of the ventricles and the peritoneal cavity, which drains the CSF. To ensure controlled CSF drainage, these shunt systems include a valve that regulates CSF flow dependent of intracranial CSF pressure.

There are many VPS valve models approved by the European Union or the FDA, and each valve model has its own way of indicating its pressure level, which is usually accomplished with a skull X‑ray. To obtain the pressure level, first the reader has to identify the respective VPS valve model at hand from the skull x‑ray or from previous medical documentation of the patient, if available. Then, the appropriate, model-specific reading template must be referenced, which shows how the specific VPS valve model indicates its pressure level and allows the interpretation of the X‑ray.

In recent years deep learning has emerged as an important corner-stone in the automatic analysis of radiologic images. Experience with similar radiological tasks, e.g., the determination of bone age from hand X‑ray images, has shown that deep learning can support the radiologist in this type of assessment by providing high accuracy and accelerating the reading process [3, 4].

A previous study showed that automatic shunt valve identification using deep learning is possible if the X‑ray image is tightly cropped around the shunt valve [5]. While this is an encouraging finding, the clinical utility of such an approach is limited. It would require manual image cropping by the reader before the deep learning model could identify the shunt valve model. Such preprocessing would not be suitable in a clinical setting and rather complicate the work of radiologists than relieving.

The goal of this study was to explore, if automated identification of VPS valve models from a skull X‑ray using a real-world dataset and without manual pre-processing steps is feasible.

This could enable the automation of this repetitive task by automatically providing the correct reading template and alleviate the increasing workload of radiologists today.

## Methods

This retrospective exploratory study was approved by the local ethics committee. The requirement for written informed consent was waived.

### Dataset

**Fig. 1:**
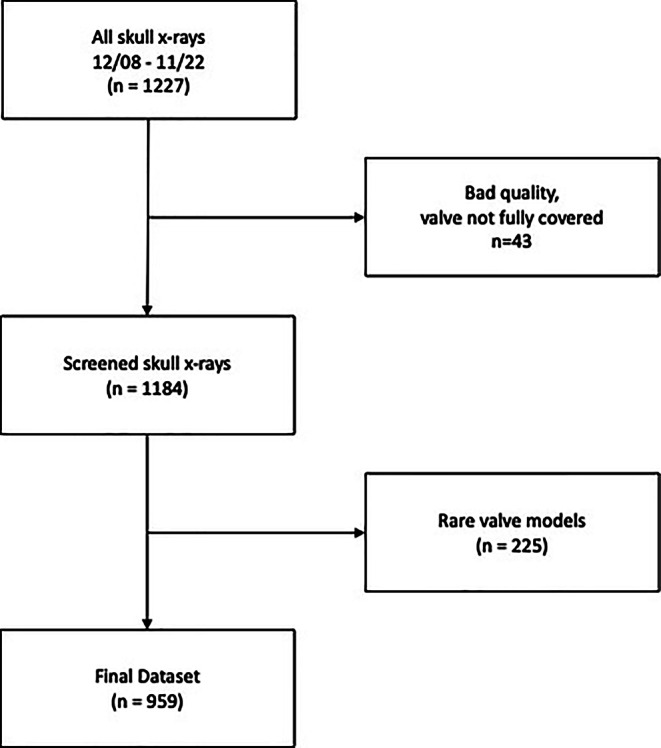
Flowchart depicting the selection process for the skull X‑rays. All skull X‑rays from 12/08–11/22 (*n* = 1227) retrieved from the PACS and screened for full valve coverage and adequate quality. In the end, 959 skull X‑rays of the four most common VPS valve models depict the final dataset

All skull x‑rays acquired between 05/08 and 11/22 at the Department of Diagnostic and Interventional Radiology of the University Hospital Düsseldorf were retrieved from the local Picture Archiving and Communication System (PACS). Only images acquired to evaluate the VPS valve were included. The images were screened for appropriate image quality and full VPS coverage. The received DICOM files were converted to JPEG using the “pydicom” package (https://github.com/pydicom/pydicom) in Python. To remove black background, the images were automatically cropped to only include pixels with a value of greater than 0 and then resized to 512 × 512 pixels using squishing. The pixel values were then normalized to a range between 0 and 1. The dataset was labeled by a radiologist with 5 years of experience (MV) by specifying the respective VPS valve model type of each x‑ray using the labeling software Label Studio (https://labelstud.io/). Only images with the four most common VPS valve models imaged at the local department (Codman Hakim, Codman Certas Plus, Sophysa Sophy SM8, proGAV 2.0) were included in the final dataset (see Fig. 1).

The resulting dataset was then split using a stratified five-fold cross-validation. Images were grouped on patient level to avoid data leakage from images of the same patient in the training and validation split.

### Model Training

A residual convolutional neural network (ResNet-34 [6], 21 million parameters) was used for image classification. ResNets are a special type of convolutional neural networks with a deep architecture and which incorporates the learning of residual functions with reference to initial layers as a characteristic feature. They are an established neural network architecture for image classification tasks and are widely used in the medical domain [7]. The neural network consists of 34 “residual layers”. A residual layer consists of two or three convolutional layers as well as a skip connection. The skip connection enables activations to flow freely through the layer without any transformation. The output of the convolutional layers will be merged with output of the skip connection, i.e., the output of the previous layer, through addition. The convolutional layers therefore only learn the residuals of the previous layer, hence the name “residual layer” [6]. It has been empirically shown that this network architecture leads to better performance as well as a more stable training process, compared to pure convolutional neural networks [6].

For the current task, the neural network was initialized with parameters from pretraining on ImageNet, a large dataset of over 1 million natural images with 1000 classes [8]. The neural network was then fine-tuned on the VPS valve classification task for 50 epochs. Additionally, a randomly initialized Resnet-34 was trained for 80 epochs from scratch, using the same training parameters.

Weighted cross entropy was used as loss function. The loss weights were calculated using the inverse of the number of cases per class in the dataset. A one-cycle learning rate policy with a maximum learning rate of 0.003 was used [9]. We used “ADAM” as the optimizer. To avoid overfitting, random data augmentation on the training split was used with the following image transformations: horizontal flipping, rotation, zoom, changing brightness and warping. All neural network training was implemented using PyTorch (version 1.12.0) and the fast.ai software library (version 2.7.10) [10]. The batch size was 32 with gradient accumulation to reach an effective batch size of 64. All training was performed on an NVIDIA Titan V GPU with 12 Gb of VRAM. Training of all five cross-validation splits took around 2 h.

### Evaluation

To evaluate the classifier accuracy, precision, recall and the macro-averaged F1-score were evaluated across all five splits of the cross-validation. All performance metrics are reported per split and as the mean and standard deviation across the cross-validation splits as well as per class averaged over all cross-validation splits. Additionally, we calculated the mean error rate over all five splits for the specific VPS valve models. To measure the uncertainty of the model, we calculated the entropy of the softmax distribution, the maximum softmax score as well as the gap between the highest and second highest softmax score. All of the uncertainty measurements were averaged over all five cross-validation splits for each class. Saliency maps showing the most relevant parts of the image for each class were created using the GradCam algorithm [11]. The data analysis was performed using Python (version 3.10.10) with the “numpy” (version 1.22.3), “PyTorch” (version 1.12.0) and “scikit-learn” (version 1.1.2) libraries.

The code for model training as well as model evaluation and analysis can be found at https://github.com/AInII-Lab/vps-dl.

## Results

### Dataset

**Fig. 2:**
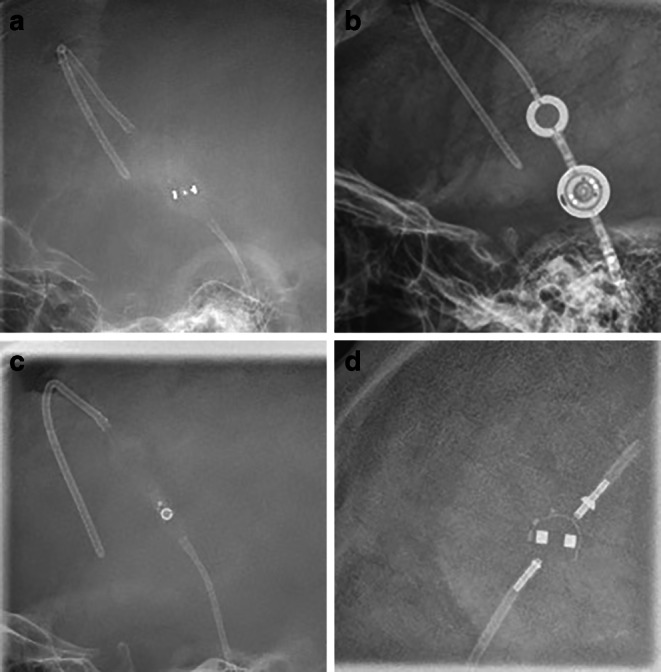
Examples of the four ventriculoperitoneal shunt (VPS) models in the final training dataset (**a** Codman Certas Plus, **b** proGAV 2.0, **c** Codman Hakim, **d** Sophysa Sophy SM 8)

The final dataset consisted of 959 skull X‑rays from 512 patients (273 (53.3%) female, age 47 ± 22.5 years) with the following VPS valve models (see Table 1): Codman Hakim (*n* = 774, 81%, Integra LifeSciences, Princeton, New Jersey), Codman Certas Plus (*n* = 117, 12%, Integra LifeSciences, Princeton, New Jersey), Sophysa Sophy Mini SM8 (*n* = 35, 4%, Sophysa, Orsay, France) and proGAV 2.0 (*n* = 33, 3%, Christoph Miethke GmbH & Co. KG, Potsdam, Germany). 212 patients (41.4%) had more than one x‑ray in the dataset. Example x‑ray images of each VPS valve model are illustrated in Fig. 2.

**Table 1:** Table of the data distribution in the final dataset (*n* = 959)

VP-Shunt Model	Count (*n* = 959) (%)
Codman Hakim (Integra LifeSciences, Princeton, New Jersey)	774 (81)
Codman Certas Plus (Integra LifeSciences, Princeton, New Jersey)	117 (12)
Sophysa Sophy SM8 (Sophysa, Orsay, France)	35 (4)
proGAV 2.0 (Christoph Miethke GmbH & Co. KG, Potsdam, Germany)	33 (3)

**Fig. 3:**
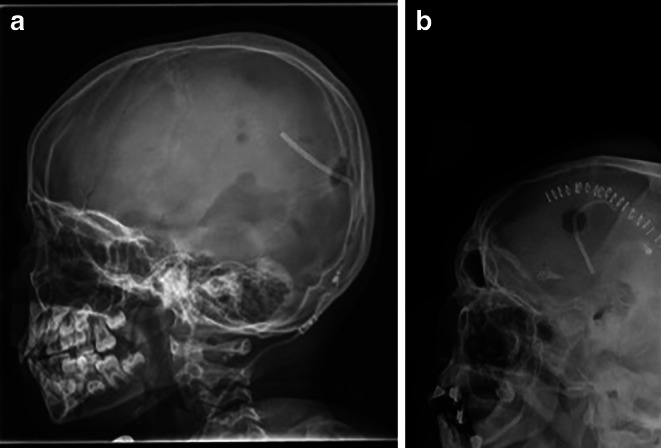
Two examples of misclassified images. The VPS valve (Codman Hakim) is at the edge of the image (**b**) or rotated (**a**) which makes it hard to identify

**Fig. 4:**
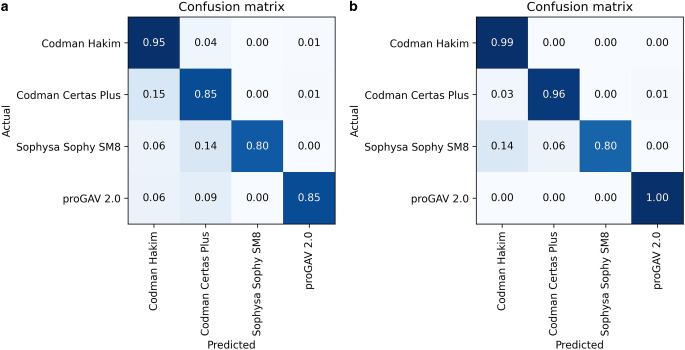
Confusion matrix normalized over all five cross-validation splits for the model without (**a**) and with (**b**) transfer learning

The convolutional neural network (CNN) that was trained from scratch reached a mean precision of 0.94 ± 0.02, a mean recall of 0.93 ± 0.02, a mean accuracy of 0.93 ± 0.03 and a macro-averaged F1 score of 0.87 ± 0.08 for the classification of VPS valve models over the five cross-validation splits (see Table 2). The mean error rate for the Codman Hakim valve was 5%, for the Codman Certas Plus valve 15%, for the Sophy SM8 valve 20% and for the proGAV 2.0 valve 15%. Two examples of misclassified valve models are shown in Fig. 3. The confusion matrix can be found in Fig. 4.

**Table 2:** Precision, recall, accuracy and macro-averaged F1 score for each cross-validation split as well as their mean over all five cross-validation splits for the model with and without transfer learning (TL)

	No TL	TL	No TL	TL	No TL	TL	No TL	TL
	Precision		Recall		Accuracy		F1Score	
Split 1	0.95	0.995	0.95	0.96	0.95	0.98	0.94	0.98
Split 2	0.92	0.99	0.91	0.91	0.91	0.99	0.80	0.94
Split 3	0.97	0.98	0.97	0.92	0.97	0.98	0.96	0.94
Split 4	0.92	0.97	0.91	0.93	0.91	0.98	0.86	0.94
Split 5	0.93	0.8	0.91	0.98	0.91	0.98	0.80	0.84
*Mean* *±* *SD*	0.94 ± 0.02	0.95 ± 0.08	0.93 ± 0.03	0.94 ± 0.03	0.93 ± 0.03	0.98 ± 0.01	0.87 ± 0.08	0.93 ± 0.4

The pretrained convolutional neural network (CNN) that was trained using transfer learning reached a mean precision of 0.95 ± 0.08, a mean recall of 0.93 ± 0.03, a mean accuracy of 0.98 ± 0.01 and a macro-averaged F1 score of 0.93 ± 0.04 for the classification of VPS valve models over the five cross-validation splits (see Table 2). The mean error rate for the Codman Hakim valve was 1%, for the Codman Certas Plus valve 4%, for the Sophy SM8 valve 20% and for the proGAV 2.0 0%.

**Fig. 5:**
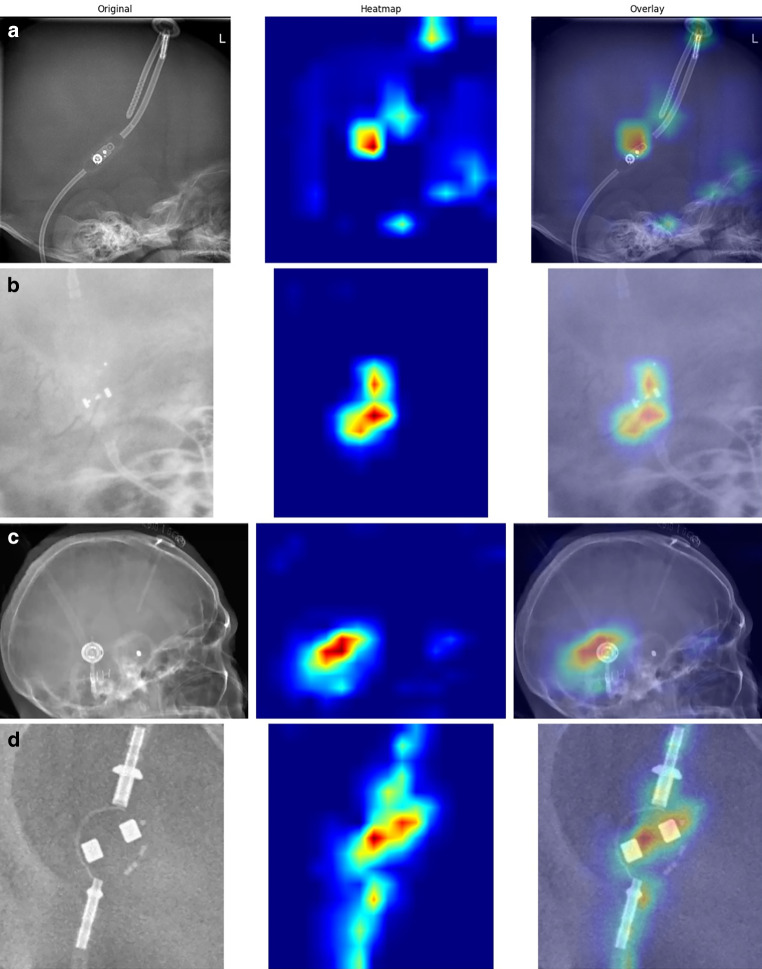
Saliency maps created with the GradCam algorithm for the four different ventriculoperitoneal shunt valve models showing the most relevant areas for each valve model (**a** Codman Hakim, **b** Codman Certas Plus, **c** Sophysa Sophy SM8, **d** proGAV 2.0)

The uncertainty measurements as well as the precision, recall and f1-score for each class can be found in Table 3. Fig. 5 shows saliency maps for each valve model. Results for a neural network trained with an additional class grouping all additional valve models can be found in the supplementary material.

**Table 3:** Precision, Recall, macro-averaged F1 Score as well as the Entropy, Max Softmax Score and the Softmax Gap for every class averaged over all 5 cross-validation splits for the model with and without transfer learning (TL)

	No TL	TL	No TL	TL	No TL	TL	No TL	TL	No TL	TL	No TL	TL
Valve Model	Precision		Recall		F1 Score		Entropy		Max Softmax Score		Softmax Gap	
Codman Hakim	0.97 ± 0.01	0.99 ± 0.01	0.95 ± 0.02	0.99 ± 0.001	0.96 ± 0.01	0.99 ± 0.001	0.24 ± 0.24	0.06 ± 0.01	0.96 ± 0.08	0.99 ± 0.001	0.93 ± 0.14	0.99 ± 0.001
Codman Certas	0.71 ± 0.12	0.96 ± 0.04	0.85 ± 0.03	0.96 ± 0.06	0.77 ± 0.08	0.96 ± 0.02	0.21 ± 0.31	0.09 ± 0.03	0.96 ± 0.09	0.98 ± 0.01	0.92 ± 0.17	0.96 ± 0.02
proGAV 2.0	1.0 ± 0.0	0.97 ± 0.06	0.84 ± 0.15	0.81 ± 0.16	0.91 ± 0.09	0.87 ± 0.08	0.09 ± 0.28	0.14 ± 0.055	0.97 ± 0.09	0.97 ± 0.02	0.95 ± 0.17	0.95 ± 0.03
Sophysa SM 8	0.91 ± 0.16	0.87 ± 0.27	0.85 ± 0.18	1.0 ± 0.0	0.85 ± 0.16	0.9 ± 0.2	0.08 ± 0.26	0.03 ± 0.021	0.98 ± 0.07	0.99 ± 0.003	0.97 ± 0.12	0.99 ± 0.01

## Discussion

The aim of this study was to explore, whether automatic identification of VPS valve models from skull x‑rays is possible using deep learning.

We successfully trained a convolutional neural network (CNN) on a set of skull x‑rays to classify the VPS valve model. We achieved promising evaluation metrics with a high accuracy and F1-score.

The accuracy of our model of 95% is comparable to a previous study which also classified VPS valve models in skull x‑rays [5]. However, the previous study applied heavy preprocessing by manually cropping the skull x‑rays around the VPS valve models. This prohibits an end-to-end use of the model, which would be beneficial, e.g., in a clinical setting.

In contrast, for the current study, x‑rays were not cropped around the VPS valve manually. Instead, images were automatically cropped, so that all black pixels along the edges of the x‑ray images were removed. After this, the model processes the whole x‑ray and is able to identify the model irrespective of the relative location of the valve within the x‑ray.

In some of the misclassified images, the VPS valve was at the edge of the image, rotated or skewed. Such constellations make it hard, even for human readers, to correctly identify the valve model. Deep learning models rely on repeated image features to identify the different valves. Therefore, it is even more challenging for the neural network to identify the valve correctly if the valve is heavily skewed and this “skewness” is not represented often in the training dataset.

Interestingly, the most common VPS valve in the dataset (Codman Hakim) as well as the least common valve (proGAV 2.0) showed the lowest mean error rates. The proGAV 2.0 looks very different compared to the other valve models, which might explain why it might be easy for the neural network to distinguish it from all other valve models, despite its low prevalence in the dataset. Additionally, it may suggest the model’s ability to classify valves irrespective of their prevalence in the dataset. Nevertheless, the difference in the error rates between the different VPS valve models also indicates potential for further improvement. Apparently, especially the model trained from scatch only shows moderate performance for the identification of the Codman Certas Plus and the Sophysa Sophy SM8 valve models. Future work could explore other neural network architectures or ensemble models to further boost performance, especially for the valve types with higher error rates.

Using a pretrained neural network as well as various data augmentation methods might explain the robust performance, even on VPS valve models with few examples in the training dataset. The technique of using a neural network pretrained on another dataset is called transfer learning [12]. With this technique the neural network learns general image features—like lines and gradients—in the pre-training dataset. On the downstream task it then only needs to “re-arrange” these low-level features to solve the actual task at hand. Our data as well as many other studies have found that this approach indeed helps with the generalization of models trained on smaller datasets, even in the medical domain, despite ImageNet consisting of natural, non-medical images [13, 14].

A potential clinical application for such deep learning model would be alleviating the valve pressure level reading process. Every VPS valve model not only has a unique way of indicating its pressure level, the pressure levels itself differ between different valves to a high degree. As a first step a deep learning model developed in a comparable fashion would enable the automated serving of the template to read the pressure level of the specific VPS valve model. In a second step another deep learning model could even detect the correct pressure level for the identified shunt valve model. Another application—that was also discussed by Giancardo et al. [5]—would be the screening for MRI compatibility of the corresponding VPS model, although most VPS valves are MRI compatible or at least MRI conditional today.

### Limitations

Our model is only capable of differentiating between four VPS valve models. The model and its real-world applicability can be improved by incorporating more training data with other VPS valve models.

Furthermore, the gravitational units often used in conjunction with the VP shunt valve, such as the ShuntAssistant (SA) with the ProGAV 2.0, which are available in different resistance classes, were not included in the analysis and were therefore not examined. The analysis focuses solely on the adjustable component of the shunt valve.

Additionally, the study was only conducted at a single imaging center with a retrospective design. An external test dataset would better assess the generalizability of our deep learning model. This is a topic for future research.

## Conclusion

To conclude, automatic classification of VPS valve models in a skull X‑ray, using fully automatable preprocessing steps and convolutional neuronal networks, is feasible. This is an encouraging finding to further explore the possibility of automating VPS valve model identification and pressure level reading in skull X‑rays.

## Supplementary Information

Results with additional “other” class

## Abbreviations

CNN: Convolutional Neural Network
CSF: Cerebrospinal Fluid
FDA: Food and Drug Administration
GPU: Graphics Processing Unit
MRI: Magnetic Resonance Imaging
PACS: Picture Archiving and Communication System
SA: ShuntAssistant
VPS: Ventroculoperitoneal Shunt
