# Supplementary material for: Deep-Learning-based Automated Identification of Ventriculoperitoneal-Shunt Valve Models from Skull X-rays
Source: Clin Neuroradiol. 2025 Jan 14;35(2):347–54. doi: 10.1007/s00062-024-01490-4 (PMC12174198; doi:10.1007/s00062-024-01490-4)
Supplement: Supplementary file 1 — Results with additional “other” class [file 62_2024_1490_MOESM1_ESM.pdf]

Results for Model with additional „other“ class

These tables and confusion matrix show the performance of a Resnet-34 model trained with transfer learning and an additional class called “other” that groups all other valve models in our dataset.

	**Precision**	**Recall**	**F1 Score**	**Entropy**	**Max SoftMax Score**	**SoftMax Gap**
**Codman Hakim**	0.97 ± 0.01	0.99 ± 0.01	0.98 ± 0.01	0.06 ± 0.01	0.99 ± 0.01	0.99 ± 0.01
**Codman Certas Plus**	0.95 ± 0.05	0.92 ± 0.05	0.93 ± 0.02	0.13 ± 0.03	0.97 ± 0.01	0.95 ± 0.02
**Other**	0.74 ± 0.1	0.75 ± 0.12	0.74 ± 0.1	0.27 ± 0.04	0.93 ± 0.01	0.89 ± 0.03
**Sophysa Sophy SM 8**	1.0 ± 0.0	0.85 ± 0.15	0.91 ± 0.09	0.22 ± 0.08	0.94 ± 0.03	0.90 ± 0.05
**proGAV 2.0**	0.91 ± 0.09	0.9 ± 0.16	0.91 ± 0.12	0.13 ± 0.04	0.97 ± 0.01	0.94 ± 0.02

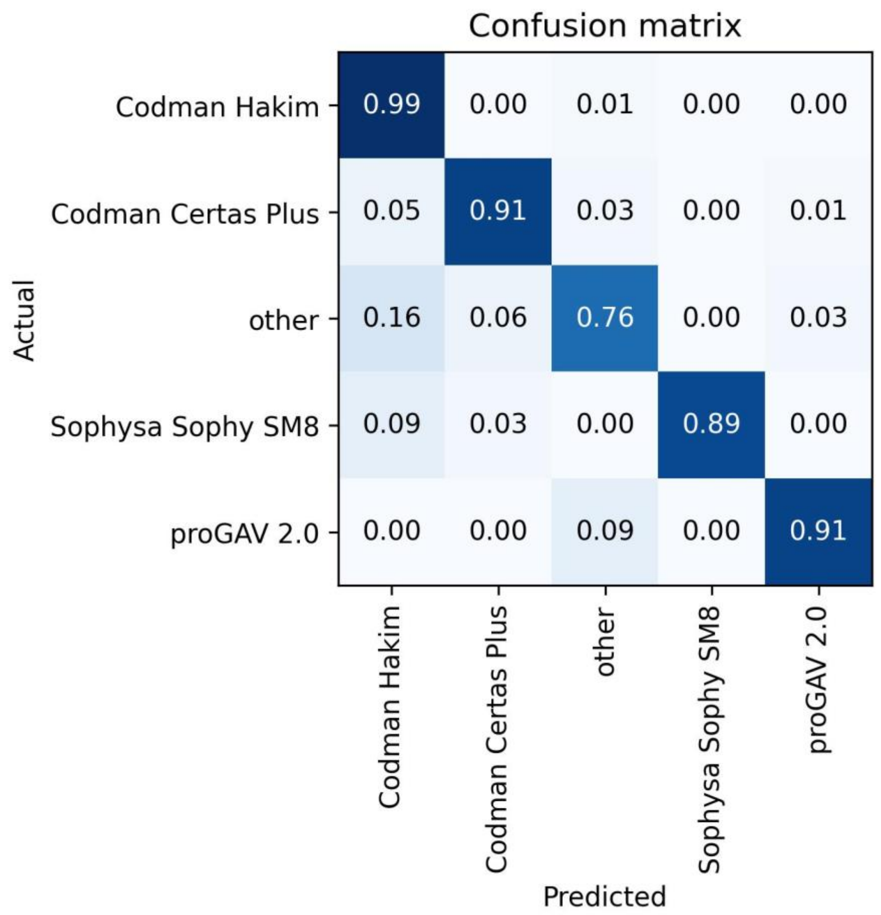

Confusion Matrix averaged over all five cross-validation splits.

**Split**	**Precision**	**Recall**	**F1 Score**	**Accuracy**
1	0.89	0.88	0.89	0.95
2	0.93	0.86	0.89	0.96
3	0.92	0.88	0.90	0.96
4	0.95	0.94	0.94	0.97
5	0.88	0.84	0.86	0.94
**Mean**	0.92 ± 0.02	0.88 ± 0.04	0.89 ± 0.03	0.96 ± 0.01

Precision, Recall, macro-averaged F1-Score as well as the accuracy for the different cross validation splits.
